# Factors associated with prior engagement in high-risk sexual behaviours among adolescents (10–19 years) in a pastoralist post-conflict community, Karamoja sub-region, North eastern Uganda

**DOI:** 10.1186/s12889-019-7352-6

**Published:** 2019-07-31

**Authors:** Rogers N. Ssebunya, Joseph K. B. Matovu, Fredrick E. Makumbi, Grace P. Kisitu, Albert Maganda, Adeodata Kekitiinwa

**Affiliations:** 1 Directorate of Research, Monitoring and Evaluation, Baylor College of Medicine Children’s Foundation, P.O. Box 72052, Kampala, Uganda; 2 Department of Disease Control and Environmental Health, School of Public Health, Makerere University College of Health Sciences, Kampala, Uganda; 3 Department of Community and Public Health, Busitema University Faculty of Health Sciences, Mbale, Uganda; 4 Department of Epidemiology and Biostatistics, School of Public Health, Makerere University College of Health Sciences, Kampala, Uganda

**Keywords:** Risk sexual behaviours, Adolescents, Conflict setting

## Abstract

**Background:**

Adolescent sexual risky behaviours continue to be significant drivers of the HIV epidemic globally. The objective of this study was to determine factors associated with prior engagement in high-risk sexual behaviours among adolescents (10–19 years) in Karamoja sub-region, a pastoralist and post-conflict community in North-eastern Uganda.

**Methods:**

Between August and September 2016, we conducted a cross-sectional study among 1439 adolescents receiving primary healthcare services at nine public health facilities located in five of the seven districts that make up Karamoja sub-region. High-risk sexual behaviour was defined as engaging in sex with two or more (2+) sexual partners in the 6 months preceding the survey or exchanging sex for money or gifts with no or inconsistent use of condoms over the same period of time. Factors associated with prior engagement in high-risk sexual behaviours were analysed using a modified Poison regression model with log-link and Poisson-family via a generalized linear model.

**Results:**

Eighty-two percent (81.8%, *n* = 1177) of the respondents had ever tested for HIV while 62 % (61.5%, *n* = 885) had ever had sex. Of those that had ever had sex, 11.4% (*n* = 101) reported prior engagement in high-risk sexual behaviours. Prior engagement in high-risk sexual behaviours was lower among men than women (adjusted prevalence ratio (adj. PR) = 0.46; 95% Confidence Interval (95% CI): 0.33, 0.62) and those whose sex debut was above 14 years (adj.PR = 0.63; 95% CI: 0.57, 0.69). However, prior engagement in high-risk sexual behaviours was significantly higher in adolescents who were not aware of their recent sexual partner’s HIV status (adj.PR = 2.43; 95% CI: 1.68, 3.52) and those who used illicit drugs (adj.PR = 2.76; 95% CI: 1.88, 4.05).

**Conclusion:**

Prior engagement in high-risk sexual behaviours was significantly associated with having sex with partners of unknown HIV sero-status and use of illicit drugs. These findings suggest a need for targeted interventions to improve mutual HIV status disclosure between sexual partners while minimizing their use of illicit drugs/substances.

**Electronic supplementary material:**

The online version of this article (10.1186/s12889-019-7352-6) contains supplementary material, which is available to authorized users.

## Background

The global fight against the HIV epidemic is pivoted on reducing sexual risky behaviours among young people, including adolescents. Despite global estimates showing a declining trend in HIV incidence in all ages, the decline among young girls and women in sub-Saharan Africa including Uganda remains pretty slow [1–4]. Adolescents and young people (10–24 years) have continued to harbour the majority of reported new HIV infections; with 42% of all new infections in 2010 [5] and 0.79 million of the global 2.1 million infections in 2015 [6] registered among adolescents 10–19 years [3]. Despite an increased vulnerability to HIV acquisition, evidence indicates that a large proportion of young people aged 15–24 years remain unaware of their HIV status [7] and continue to engage in risky sexual behaviours [8, 9]. Additionally inadequate HIV knowledge, lack of access to treatment and poor adherence to medication among adolescents [10, 11], among other factors, further taint this blurred picture in the struggle to end AIDS by 2030. Further evidence alludes to even worse indicators in conflict and pastoralist communities [12–14]. Crises due to conflicts have been documented to increase people’s vulnerability to HIV/AIDS acquisition especially among girls and women [15, 16]. With 80% of the global adolescents living with HIV/AIDS residing in sub-Saharan Africa [17, 18], adolescents in post-conflict and pastoralist communities present a double-barrelled challenge in the fight against the epidemic.

The attainment of global HIV targets requires a holistic understanding of context-specific adolescent sexual and behavioural characteristics. The 2014 UNAIDS gap report [19], the 2016 UNAIDS fast-track report on commitments to end AIDS [20], and other reports [21, 22] have all highlighted the need to refocus HIV prevention efforts on the most at-risk populations including adolescents, if we are to reach the 2020 targets. Determinants for engagement in risky sexual behaviours have been documented elsewhere in varying populations [23–28]. The 2014 WHO report on adolescent health elucidated various forces behind adolescent health behaviour including individual factors like age, sex and knowledge, family and community factors, cultural practices and norms [29].

Despite existing evidence, few or virtually no study has documented about adolescents’ engagement in high-risk sexual behaviours in post-conflict and pastoral settings and yet such settings present a unique challenge in accessing basic health care services including HIV/AIDS services, complicate sexual decision-making and increase vulnerability for HIV infection [16]. Individuals from mobile communities like the one we focus on in this study have been found to be more likely to fall out of the routine HIV surveillance system. This was underscored in the UNAIDS guidelines for HIV/AIDS interventions in humanitarian/ emergency settings [30]. Additionally, prior studies have largely focused on young people 15–24 years but not on those aged 10–19 years thus presenting a missed opportunity for targeting this vulnerable yet HIV risk-prone age-group. This study therefore aimed to assess the factors associated with prior engagement in high-risk sexual behaviours among adolescents (10–19 years) in Karamoja sub-region, a pastoralist and post-conflict community in Uganda.

## Methods

### Study setting

The study was conducted at nine public health facilities in five of the seven districts (Moroto, Kotido, Abim, Kaabong and Napak) in Karamoja sub-region, North-eastern Uganda. Karamoja, one of Uganda’s poorest sub-regions [31] is inhabited by culturally nomadic people where boys and men look after cattle while girls do the housework. This situation is, however, changing slowly as more young men and women now spend much of their time in search for gold and other minerals. Often times these mining activities occur day and night with no clear by-laws regulating the practice. Karamoja sub-region has a population of about 988,429 people with 50% of these under the age of 18 years [32]. A large proportion (60.4%) of young people in this sub-region are sexually active and engage in early marriages; marrying as early as 9 years [33]. Close to 24 % (23.6%) of women aged 15–19 years have already given birth [34]. HIV prevalence in North-eastern Uganda, where Karamoja sub-region is located, has tripled from 1.7% in 2000 to 5.3% in 2011 [35]. Because of tribal conflicts over the years, access to education and health services including information on HIV/AIDS and reproductive health has been a challenge [12, 31, 33]. Additionally, cultural practices such as wife inheritance (42.2%), tattooing (31.8%) and female genital mutilation (23.4%) [33] continue to increase the risk of HIV acquisition among adolescents in this sub-region.

### Study design and population

This was a cross-sectional study conducted among 1439 adolescents aged 10–19 years receiving primary health care services at all eligible entry points in the above-mentioned health facilities between August and September 2016. Entry points included; out-patient department (OPD), antenatal care, post-natal care and youth corners. Only adolescents who provided written informed consent and/or assented to participate in the study were included in this study. Severely ill adolescents and those with emergency conditions such as accidents were excluded.

### Sample size estimation

We used Bennett’s formula [36] to estimate the sample size for this study. Assuming a design effect (D) of 2.0, a conservative 50% prevalence (p) of HIV knowledge among adolescents and a variance (s) of +/− 2% (0.02) in HIV knowledge among adolescents, and a non-response of 15%, we determined that we would need to select nine clusters (facilities) and a minimum sample size of 1440 respondents, considering that each health facility served approximately 140–160 adolescents on a daily basis. In this study we interviewed 1449 adolescents but dropped 10 that had missing data on two variables; condom use and transactional sex.

### Sampling

To identify which nine health facilities to select, we purposively sampled facilities that had contributed 80% of adolescents at the out-patient department (OPD) in the previous year. The 80% mark was chosen to allow an adequate representation of adolescents within the entire sub-region to be included in the study. Nine health facilities (Moroto regional referral hospital, Matany, Kaabong and Abim hospitals, Karenga and Kotido HC IV and Lokopo, Panyangara and Rengen HC III) in five out of the seven districts were selected. The five selected districts were Abim, Kaabong, Kotido, Moroto and Napak. Using the number of adolescents who turned up for HIV testing services (HTS) in the quarter prior to data collection (April–June 2016), probability proportional to size (PPS) sampling was applied to determine the number of adolescents to be interviewed per facility. Within each facility entry point adolescents were consecutively sampled until the required number per facility was obtained.

### Data collection procedures

Data were collected between August and September 2016 using an interviewer-administered, paper-based, semi-structured questionnaire and a modified questionnaire (attached as Additional file 1) adapted from a validated HIV knowledge tool (HIV KQ-18) [37] whose higher scores were correlated with adequate knowledge of HIV. We modified the questionnaire to suite the sub-region’s cultural context. For example, item 3, 8, 12, 16, 17 and 18 in the original questionnaire were paraphrased or removed to suit local cultural context and understanding. Interviews were done by trained data collectors well acquainted with the local languages to ease the process of data collection. Data quality assurance and control were done at different levels; at health facility, regional and data compilation point (central office). Specifically, quality control was done by data collectors who checked all fields in the completed questionnaires in real time before participants left the facility while data quality assurance was done by the field supervisors who reviewed all completed questionnaires submitted to them on a daily basis before filing them. Data were collected on socio-demographic characteristics, sexual risky behaviours, knowledge of HIV and HIV status awareness (i.e. ever tested for HIV and received one’s HIV test results), use of illicit drugs/substances, including alcohol before sex; age at first sex debut, and reported life time number of children ever had. After data collection had been completed, questionnaires were entered into a Microsoft Access database developed specifically for this survey in readiness for analysis.

### Measurement variables

Adolescents were considered to have engaged in ‘high-risk sexual behaviour’ if they reported to have engaged in sex with two or more (2+) partners in the 6 months preceding the survey or exchanged sex for money or gifts with no or inconsistent condom use over the same period of time. This was the main outcome of the study and computed as a composite variable based on three independent variables; reporting of two or more sexual partners in the past 6 months, exchanging sex for money or gifts (i.e. transactional sex) and/or reporting no or inconsistent condom use. Adolescents were asked to state how many sexual partners they had had within 3–6 months preceding the survey and those who reported two or more sexual partners were considered to have had multiple sexual partners. Adolescents who reported that they have ever had sex in exchange for an incentive of any nature including money were considered to have engaged in transactional sex. Those who used the condom occasionally in their last sexual encounters were considered to have inconsistently used them. The variable, ‘high-risk sexual behaviour’, was categorized as a binary outcome with one (1) representing “Yes” and zero (0) representing “No”. Level of HIV knowledge was assessed based on an aggregate score; with adolescents scoring more than the median score (> 11) considered to have adequate HIV knowledge.

### Statistical analysis

Descriptive statistics for both HIV knowledge and sexual risky behaviours were presented as frequencies and percentages. Differences in categorical variables were tested using a Chi-square test while a two-sample t-test was used for the continuous variables. Characteristics of adolescents with risky sexual behaviours were compared by sex and age-group and were descriptively presented. At the bivariate analysis, we simultaneously analysed the association between each of the independent variables and high-risk sexual behaviours while assessing their level of significance. All variables with a *p*-value of less or equal to 0.2 at the bivariate analysis (sex, marital status, use of substances or illicit drugs, sex debut, HIV status awareness) and suspected confounders (age group, occupation, level of education, number of children, alcohol use and HIV knowledge) were entered into the multivariable model using a forward stepwise approach to determine the prevalence ratios associated with prior engagement in high-risk sexual behaviours. Prevalence ratios (PRs) were generated via a modified Poison regression model with log-link and Poisson-family to determine factors independently associated with prior engagement in high-risk sexual behaviours. The regression models accounted for clustering at the health facility where participant enrolment occurred. Analyses of sexual risky behaviours were restricted to sexually active adolescents (respondents who reported that they had ever had sexual intercourse). Akaike’s information criterion was used to assess for model parsimony. All analyses were conducted using STATA version 13. We report our findings in line with the recommended “Strengthening of Observational Studies in Epidemiology” (STROBE) statement guidelines [38].

### Ethical considerations

All adolescents who were eligible to provide their own consent (i.e. aged 18 years or older) were asked to provide written informed consent in fulfilment of the ethical requirement by the ethics committee that approved this study. Adolescents who were below 18 years of age (and who, by Ugandan laws, are considered as ‘children’ who cannot provide their own consent to participate in the study) were interviewed only after obtaining parental/guardian consent (some adolescents came to the clinic with their parents or guardians, so it was easy to meet with them and explain the purpose of the study) followed by the adolescents’ informed assent. Adolescents aged 10–17 years who did not come with a parent/guardian were encouraged to talk to their parents/guardians about the purpose of the study and the need for parental/guardian consent at the clinic. Only those who came back with their parents or guardians within the data collection period were enrolled in the study after obtaining parental/guardian consent and the adolescent’s informed assent. Both consent and assent forms were translated into local languages to ease comprehension by the respondents.

## Results

### Respondents’ socio-demographic characteristics

Table 1 shows the characteristics of adolescents that were enrolled into this study. Of the 1439 adolescents enrolled, 62.8% (*n* = 904) were young women while 83.6% (*n* = 1203) were aged 15–19 years. A significantly higher proportion of young women than men were married (young women: 48.3% vs. young men 16.6%, *p* = 0.001). On the other hand, a large proportion of male adolescents were in school (young men: 68.0% vs. young women 41.3%, *p* = 0.001), more educated (young men: 38.3% vs. young women 29.0%, *p* = 0.001) and with adequate level of knowledge on HIV prevention and transmission than female adolescents (young men: 51.2% vs. young women 43.8%, *p* = 0.001).

**Table 1:** Socio-demographic characteristics of adolescents

Characteristics		Young men, *n* (%)	Young women, *n* (%)	Total
All		535 (37.2)	904 (62.8)	1439
Age group	10–14	104 (19.4)	132 (14.6)	236 (16.4)
	15–19	431 (80.6)	772 (85.4)	1203 (83.6)
Marital status	Currently married/Cohabiting	89 (16.6)	437 (48.3)	526 (36.6)
	Currently not married	446 (83.4)	467 (51.7)	913 (63.5)
Education	No education/ECD	78 (14.6)	276 (30.5)	354 (24.6)
	Primary / ABEK^a^	252 (47.1)	366 (40.5)	618 (43.0)
	≥ Secondary	205 (38.3)	262 (29.0)	467 (32.5)
Occupation	Student	364 (68.0)	373 (41.3)	737 (51.2)
	Cattle keeper	77 (14.4)	6 (0.7)	83 (5.8)
	None/ Other^b^	94 (17.6)	525 (58.0)	619 (43.0)
Health facility level	Health centre III	87 (16.3)	200 (22.1)	287 (19.9)
	Health centre IV	99 (18.5)	177 (19.6)	276 (19.2)
	District general hospital	118 (22.1)	208 (23.0)	326 (22.7)
	Regional referral hospital	231 (43.2)	319 (35.3)	550 (38.2)
Adequate HIV knowledge	Yes	274 (51.2)	387 (43.9)	671 (46.6)
	No	261 (48.8)	507 (56.1)	768 (53.4)
District of residence	Abim	41 (7.7)	111 (12.3)	152 (10.6)
	Kaabong	82 (15.3)	122 (13.5)	204 (14.2)
	Kotido	130 (24.3)	279 (30.9)	409 (28.4)
	Moroto	232 (43.4)	317 (35.1)	549 (38.2)
	Napak	50 (9.4)	75 (8.3)	125 (8.7)

^a^ *ABEK* Alternative Basic Education for Karamoja, ^b^ Farmer, business person, Boda boda, firewood seller, *ECD* Early Childhood Development

### Adolescents’ sexual behaviours by individual characteristics

Table 2 shows the distribution of adolescents’ sexual behaviour by sex and age. Nearly 62 % (*n* = 885) of adolescents have ever had sex, with an overall median (IQR) age at sex-debut of 15 years (range: 14 to 16) years. Majority (*n* = 867, 98%) of those that have ever had sex were aged between 15 and 19 years while 611 (69.0%) were young women. Of the sexually active adolescents, 134 (15.1%) reported that they had engaged in sex with multiple sexual partners [young men: 74 (27.0%) vs. young women: 60 (9.8%), *p* = 0.001]. Only 21 (2.4%) of the adolescents reported that they had ever engaged in transactional sex; with a slightly higher proportion of young men than women reporting this sexual practice [men: 9 (3.3%) vs. women: 12 (2.0%), *p* = 0.233]. Inconsistent condom use was high (82.2%, *n* = 727), with more young women than men reporting this behaviour [women: 530(86.7%) vs. men: 197(71.9)]. Additionally, a higher proportion of young women were aware of their most recent sexual partner’s HIV status than their male counterparts [young women: 421(68.9% vs. men: 163 (59.5%), *p* = 0.001]. There was no significant difference between young men and women with regard to mean age at sex debut (*p* = 0.618).

**Table 2:** Sexual behaviours by respondent’s sex and age category

Variable		Sex of respondent		*P*-value	Age category		
All		Male, *n* (%)	Female, *n* (%)		10–14, *n* (%)	15–19, *n* (%)	*p*-value
		*N* = 535 (37.2)	904 (62.8)		236 (16.4)	1203 (83.6)	
Ever had sex^a^	Yes	274 (51.2)	611 (67.6)	0.001^b^	18 (7.6)	867 (72.1)	0.001^b^
	No	261 (48.8)	293 (32.4)		218 (92.4)	336 (27.9)	
Condom use^b^	Always	77 (28.1)	81 (13.2)	0.001^b^	3 (16.7)	155 (17.9)	0.989
	Sometimes	83 (30.3)	116 (19.0)		4 (22.2)	195 (22.5)	
	Not at all	114 (41.6)	414 (67.8)		11 (61.1)	517 (59.6)	
Multiple sexual partners^b^	Yes	74 (27.0)	60 (9.8)	0.001^b^	3 (16.7)	131 (15.1)	0.855
	No	200 (73.0)	551 (90.2)		15 (83.3)	736 (84.9)	
Awareness of partner’s HIV-status^b^	Yes	163 (59.5)	421 (68.9)	0.006^b^	4 (22.2)	580 (66.9)	0.001^b^
	No	111 (40.5)	190 (31.1)		14 (77.8)	287 (33.1)	
Transactional Sex^b^	Yes	9 (3.3)	12 (2.0)	0.233	0 (0)	21 (2.4)	0.504
	No	265 (96.7)	599 (98.0)		18 (100)	846 (97.6)	
Age of sex debut, mean(SD)^b^		14.9 (2.1)	15.0 (2.3)	0.618	11.9 (1.41)	15.1 (2.21)	0.001^b^

^a^ Among all interviewed (*n* = 1439), ^b^ Among those who have ever had sex (*n* = 885)

Table 3 shows the distribution of prior engagement in high-risk sexual behaviors by adolescents’ individual characteristics. Overall, 101 (11.4%) of all sexually active adolescents aged 10–19 years reported that they had ever engaged in high-risk sexual behaviours. A higher proportion of young men reported prior engagement in high-risk behaviours than women (20.1% vs. 7.5%, *p* = 0.001) as were adolescents who were not currently married compared to those that were married (14.4% vs. 9.4%, *p* = 0.022). Higher levels of prior engagement in high-risk sexual behaviours were reported by adolescents aged 10–14 years than their 15–19 year-old counterparts (16.7% vs. 11.3%, *p* = 0.479). Table 3 also shows that a large proportion of adolescents who didn’t know their partners’ HIV status (19.3% vs. 7.4%, *p* = 0.001) and those who used illicit drugs/substances (41.2% vs. 10.8%, *p* = 0.001) were significantly more likely to report prior engagement in high-risk sexual behaviours than their counterparts. Additionally, adolescents with a higher level of formal education reported to have ever engaged in high-risk sexual behaviours than their less-educated counterparts [≥ secondary (13.2%) vs. primary education (12.9%) vs. no education (7.0%), *p* = 0.042].

**Table 3:** Distribution of prior engagement in high-risk sexual behaviours among adolescents in a pastoralist, post-conflict setting in Uganda by background characteristics

Variable		Risky sexual behaviours		
All	*N* = 885	Yes, *n* (%)	No, *n* (%)	*p*-value
		101 (11.4)	784 (88.6)	
Sex	Male	55 (20.1)	219 (79.9)	0.001^a^
	Female	46 (7.5)	565 (92.5)	
Age group	10–14	3 (16.7)	15 (83.3)	0.479
	15–19	98 (11.3)	769 (88.7)	
Marital status	Currently married / cohabiting	49 (9.4)	474 (90.6)	0.022^a^
	Not currently married	52 (14.4)	310 (85.6)	
Education	No education /ECD	17 (7.0)	225 (93.0)	0.042^a^
	Primary /ABEK	36 (12.9)	244 (87.1)	
	≥ Secondary	48 (13.2)	315 (86.8)	
Occupation	None & Others	53 (10.5)	452 (89.5)	0.333
	Student/ Pupil	44 (13.3)	286 (86.7)	
	Cattle keeper	4 (8.0)	46 (92.0)	
Knew partner HIV status	Yes	43 (7.4)	541 (92.6)	0.001^a^
	No	58 (19.3)	243 (80.7)	
No of children ever had	None	63 (12.0)	463 (88.0)	0.522
	1–3 children	38 (10.6)	321 (89.4)	
Ever tested for HIV	Yes	86 (10.8)	714 (89.2)	0.057
	No	15 (17.7)	70 (82.3)	
Use of drugs/ substances^b^	Yes	7 (41.2)	10 (58.8)	0.001^a^
	No	94 (10.8)	774 (89.2)	

^a^Either engaged in transactional sex or multiple sexual partners PLUS inconsistent condom use, ^b^ Marijuana, Kuber, Tobacco smoking

### Correlates of prior engagement in high-risk sexual behaviours among adolescents

Table 4 shows the factors associated with prior engagement in high-risk sexual behaviours among adolescents aged 10–19 years. At the bivariate analysis, the factors that were significantly associated with prior engagement in high-risk sexual behaviours were; sex, marital status, level of education, knowledge of partner’s HIV status, age of sex debut and use of substances/illicit drugs. In the final multivariable model, the factors that were independently associated with prior engagement in high-risk sexual behaviours included; not knowing the HIV status of their previous sexual partner, illicit use of drugs/substances, being female and age at sexual initiation. Specifically, the probability of reporting prior engagement in high-risk sexual behaviours was significantly higher among adolescents who never knew their most recent sexual partner’s HIV status than those who knew [adjusted prevalence ratio (adj.PR) = 2.43, 95% Confidence Interval (95% CI): 1.68, 3.52, *p* = 0.001)]. Similarly, adolescents who reported to have used illicit drugs/substances were 2.76 times more likely to report prior engagement in high-risk sexual behaviours than those who didn’t [adj.PR = 2.76 (95% CI: 1.88, 4.05, *p* = 0.001)]. We also found that young women had a lower probability of reporting prior engagement in high-risk sexual behaviours than their male counterparts [adj.PR = 0.46 (95% CI: 0.33, 0.62, *p* = 0.001)]. Adolescents who initiated sex at or after age of 15 years were significantly less likely to report prior engagement in high-risk sexual behaviours than those who initiated sex at an early age [adj.PR = 0.63 (95% CI: 0.57, 0.69, *p* = 0.001)]. Level of education, being knowledgeable about HIV prevention and transmission, marital status and having been tested before were all not significantly associated with prior engagement in high-risk sexual behaviours in this setting.

**Table 4:** Crude and adjusted prevalence ratios associated with prior engagement in high-risk sexual behaviours among adolescents in a pastoralist, post-conflict setting in Uganda

Variable		Risky sexual behaviour^π^, *n* = 101	Percent (%)	Unadj.PR^¶^	95% CI	adj.PR^¶^	95% CI	*p*-value
Sex	Male	55	20.1	1.0 (ref)	0.27–0.52	0.46	0.33–0.62	0.001^a^
	Female	46	7.5	0 .38				
Marital status	Currently married or cohabiting	49	9.4	1.0 (ref)	0.79–2.99	0.81	0.33–1.99	0.643
	Currently not married	52	14.4	1.53				
Knew partners’ sero-status	Yes	43	7.4	1.0 (ref)	2.0–3.43	2.43	1.68–3.52	0.001^a^
	No	58	19.3	2.62				
Ever tested for HIV	Yes	86	10.8	1.0 (ref)	1.35–1.99	1.16	0.98–1.36	0.078
	No	15	17.7	1.64				
Age of sex debut	10–14	43	15.9	1.0 (ref)	0.43–0.80	0.63	0.57–0.69	0.001^a^
	15–19	58	9.4	0.58				
Education level	No Education/ECD	17	7.0	1.0 (ref)				
	Primary / ABEK	36	12.9	1.83	0.77–4.34	1.51	0.60–3.82	0.379
	≥ Secondary	48	13.2	1.88	0.74–4.78	1.49	0.51–4.34	0.460
Adequate HIV knowledge	No	43	10.2	1.0 (ref)	0.95–1.60	1.15	0.72–1.86	0.556
	Yes	58	12.6	1.23				
Use of drugs/ substances	No	94	10.8	1.0 (ref)	2.71–5.34	2.76	1.88–4.05	0.001^a^
	Yes	7	41.2	3.80				

^a^Significance level < 0.05, ^*¶*^*PR* Prevalence ratio

## Discussion

Our study that aimed to assess correlates of prior engagement in high-risk sexual behaviours among adolescents (10–19 years) in a pastoralist, post-conflict community, shows that only 11.4% of adolescents reported that they had ever engaged in high-risk sexual behaviours. Adolescents who didn’t know their most recent sexual partner’s HIV status and those who used illicit drugs/substances were significantly more likely to report prior engagement in high-risk behaviours than their counterparts. We also found that young women were less likely to report prior engagement in high-risk sexual behaviours than their male counterparts as were adolescents who reported that they initiated sex at or after the age of 15 years. These findings suggest a need for adolescent-targeted interventions to improve mutual HIV status disclosure between sexual partners while minimizing their use of illicit drugs/substances.

The finding that 11.4% of the adolescents reported prior engagement in high-risk sexual behaviours is not surprising considering the historical arrangement of this society (enclosed family settings, no urbanisation, more engaged in cattle rearing). However, while 11.4% can be considered to be a smaller proportion of adolescents engaging in high-risk sexual behaviours compared to findings from other studies [39–41], the fact that the Karamoja sub-region has experienced increasing HIV prevalence since 2000 [35] raises serious public health concerns and suggests a need for more targeted risk-reduction interventions for adolescents aged 10–19 years in this setting. For instance, the probability that HIV can spread from the few adolescents who engage in high-risk behaviours to other adolescents in the community remains high as adolescents from diverse settings interact during times of courtship and eventually marriage. Besides the socio-cultural and economic dynamics of the Karamojong people, the existing high proportion of sexually active young people, those out of school [33], early and forced girl child marriage, wife inheritance, high unemployment [42] and youth migration from other parts of the country in search for gold can potentially increase the likelihood of HIV transmission in this hitherto closed society.

Our study shows a higher probability of prior engagement in high-risk sexual behaviours among adolescents who were unaware of their most recent sexual partners’ HIV status. This finding has implications for HIV transmission among adolescents especially if one of the partners was already infected with HIV and calls for a need to promote mutual HIV status disclosure between sexual partners. Besides, it is likely that adolescents who engage in high-risk sexual behaviours in this setting are not empowered to bargain and demand for HIV status disclosure from their peers before they engage in sex with them. This calls for a need to empower adolescents to request that their sexual partners disclose their HIV status to them or (if they fail to do so), the need to strengthen adolescents’ condom negotiation skills and the ability to refuse sex in the event that a condom is not used by a partner whose HIV status is not known [43].

Adolescents who used illicit drugs or substances were almost 3 times more likely to engage in risky sexual behaviours compared to those who did not use them. Use of illicit substances/drugs in this initially rural semi-arid part of the country was rather a surprising finding given that substance abuse has been more common in urban settings [44, 45]. The finding that adolescents who used illicit drugs/substances were more likely to report prior engagement in high-risk sexual behaviours was expected because of the known linkage between substance use and engagement in high-risk sexual behaviours [46–48]. With the increasing trends in urbanisation coupled with the discovery of gold in Karamoja sub-region, the probability that adolescents will further engage in high-risk sexual behaviours cannot be ruled out. As noted, the discovery of gold in Karamoja sub-region is already creating an influx of people from other districts who come in to search for gold.

As more and more people come to Karamoja, it is likely that the safety nets that once protected the Karamojong youths from risky sexual behaviours will no longer hold and this might result in young people copying risky behaviours from immigrants, including use of illicit drugs or engagement in multiple sexual partnerships. However, these are just possibilities. Our study did not attempt to explore the association between the discovery of gold and high-risk behaviours or use of illicit drugs or other substances. Further research is needed to understand the impact that the discovery of gold in Karamoja sub-region and the continued use of illicit drugs/substances will have on sexual-risk taking behaviours among adolescents in the future. Future studies should also explore the likely impact of these behaviours on HIV incidence in this setting where HIV prevalence has already increased by three-fold since 2000.

Female adolescents in our study were less likely to have engaged in prior risky sexual behaviours than their male counterparts. Because of early focused marriages [42] coupled with the high HIV prevalence among female adolescents [49], this was rather an unexpected finding. However, the finding that female adolescents were less likely to engage in high-risk behaviours could reflect a cultural norm – where women under-report the likelihood of their engagement in high-risk sexual behaviours while men exaggerate their engagement in similar behaviours [50]. On the other hand, this finding could reflect the existing division of labour in Karamoja sub-region where men look after animals while women do the housework and because of such diversity in gender roles, male adolescents tend to have more opportunities to engage in multiple sexual partners outside the home compared to their female counterparts. However, previous studies have yielded conflicting findings on this subject. In Vietnam and Kenya, for instance, studies show that male adolescents were more likely to engage in risky sexual behaviours than their female counterparts [Vietnam (52% for men, 4% for women) and Kenya (98% for young men, 56% for young women)] [51]. Yet, in a multisite study among adolescents receiving mental health treatment, women were found to be 2.6 times more likely to engage in risky sexual behaviours (inconsistent condom use) than their male counterparts [52].

Collectively, our findings and those from other studies, suggest a need to target both adolescent girls and boys in HIV prevention programs. This is especially important considering that HIV prevalence in Karamoja sub-region where the study was conducted has increased from 3.5% in 2006 to 5.3% in 2011 [35] and 3.7% in 2016 [53] with regional reports indicating a further increase by 0.3% by the end of 2017. Thus, while majority of the adolescent girls enrolled in our study did not engage in high-risk sexual behaviours, they live in a setting with increasing HIV prevalence which necessitates a need for more targeted HIV prevention interventions to reduce their risk of getting infected with HIV.

Our finding that adolescents who initiated sex at age 15 or older were less likely to engage in high-risk sexual behaviours illustrates the importance of keeping adolescents in school since evidence shows that in-school adolescents tend to delay sexual initiation and engage in less risky sexual behaviours [54–56]. Early sex initiation among young people including adolescents has been shown to predispose them to a myriad of sexual and reproductive health complications including HIV acquisition [57]. With existing evidence [58, 59] indicating decreasing condom use among adolescent girls and boys in general, organizations implementing HIV prevention programs should focus on interventions aimed at delaying sexual initiation in order to reduce the risk of HIV acquisition among adolescents and young people in this setting.

### Study limitations and strengths

Our study had some limitations. First and foremost, HIV knowledge was assessed using an adapted tool (KQ-18 questionnaire) which was modified to suit the cultural context. This might have reduced its content validity. We, however, kept most of the items in the original tool; so, we think that the modified questionnaire retained its content validity. Nevertheless, we recommend further research to assess the psychometric properties of the questionnaire used to assess HIV knowledge in this setting among the same population. Secondly, we consecutively sampled adolescents who turned up in selected health facilities and those whose guardians had provided parental consent. We acknowledge that adolescents who did not attend health services within the study period could have been more likely to engage in risky sexual behaviours than those in our study and this could have under estimated and biased the level of risk as presented in this study. These results might not therefore be generalizable to other post conflict settings. Further research is therefore warranted to reach more adolescents in such settings, and there is a need for research to document the impact that the discovery of gold in the area will have on sexual risk-taking behaviours in the future as more and more people come to live and work with adolescents in this hitherto closed society.

Despite the above-mentioned limitations, we believe that our study had a number of strengths, too. For instance, to the best of our knowledge, this is the first study to assess prior engagement in high-risk behaviours among Ugandan adolescents in such settings based on a composite definition of risky sexual behaviours. The other adolescent-based studies were conducted in other settings other than pastoralist, post conflict settings and assessed risk based on individual characteristics such as multiple sexual partners, inconsistent condom use or early sex debut. Our findings therefore provide critical data on the local setting dynamics that are needed to inform the design of HIV prevention interventions to mitigate the burden of HIV among young people in such settings.

## Conclusion

Prior engagement in high-risk sexual behaviours was significantly associated with having sex with partners of unknown HIV sero-status and use of illicit drugs. These findings suggest a need for targeted interventions to increase knowledge of partners’ HIV status and reduce illicit use of drugs among adolescents in this setting.

## Additional files

Additional file 1: Modified HIV knowledge assessment tool. (PDF 208 kb)

## Data Availability

The datasets used and/or analysed during the current study are available from the corresponding author on reasonable request.

## Abbreviations

ABEK: Alternative Basic Education for Karamoja
AIDS: Acquired Immunodeficiency Syndrome
AMICAALL: Alliance of Mayors on Health and Leaders on HIV & AIDS in Africa
ANC: Antenatal Care
ECD: Early Childhood Development
HIV: Human immunodeficiency virus
NCST: National Council for Science and Technology
OPD: Out-patient department
OVC: Orphans and Vulnerable Children
PNC: Postnatal Care
PR: Prevalence ratio
