# Supplementary material for: Factors associated with prior engagement in high-risk sexual behaviours among adolescents (10–19 years) in a pastoralist post-conflict community, Karamoja sub-region, North eastern Uganda
Source: BMC Public Health. 2019 Jul 31;19:1027. doi: 10.1186/s12889-019-7352-6 (PMC6670181; doi:10.1186/s12889-019-7352-6)
Supplement: Supplementary file 1 — Modified HIV knowledge assessment tool. (PDF 208 kb) [file 12889_2019_7352_MOESM1_ESM.pdf]

Supplementary Material I: Modified HIV knowledge assessment tool

For each statement, please circle “True” (T), “False” (F), or “I don’t know” (DK). If you do not know, please do not guess, please circle “DK”.

**Qn**	**Item**	**True**	**False**	**I don’t know**
1	Coughing and sneezing DO NOT spread HIV.	T	F	DK
2	A person can get HIV by sharing a cup of water or a meal with someone who has HIV.	T	F	DK
3	Washing private parts after sex keeps a person from getting HIV	T	F	DK
4	All pregnant women infected with HIV will have babies born with HIV	T	F	DK
5	People who have been infected with HIV quickly show serious signs of being infected	T	F	DK
6	There is medicine that can cure HIV	T	F	DK
7	A woman can NOT get HIV if she has sex with an infected person during her periods	T	F	DK
8	There is a female condom that can help decrease a woman’s chance of getting HIV	T	F	DK
9	Using a condom can prevent someone from getting HIV	T	F	DK
10	Having sex with more than one partner can increase a person’s chance of being infected with HIV.	T	F	DK
11	Taking a test for HIV one week after having sex will tell a person if she or he has HIV.	T	F	DK
12	A person can get HIV by sharing a latrine, bathroom with a person who has HIV	T	F	DK
13	Being circumcised decreases the chances of getting HIV	T	F	DK
14	There is medicine that can help someone with HIV to live longer	T	F	DK

***Source:**Modified tool guided by Carey&Schroder (2002), HIV-KQ-18. Internal consistency across samples (alphas = 0.75 to 0.89).
